# Dietary fish oil supplement induces age-specific contractile and proteomic responses in muscles of male rats

**DOI:** 10.1186/s12944-020-01333-4

**Published:** 2020-07-09

**Authors:** David W. Russ, Kalina Dimova, Emily Morris, Marguerite Pacheco, Sean M. Garvey, Stylianos P. Scordilis

**Affiliations:** 1 School of Physical Therapy & Rehabilitation Sciences, University of South Florida, 12901 Bruce B. Downs Blvd., Tampa, FL MDC77 USA; 2 Ohio Musculoskeletal and Neurological Institute (OMNI), Heritage College of Osteopathic Medicine, Athens, OH USA; 3 Center for Proteomics, Smith College, Northampton, MA USA; 4 Program in Biochemistry, Smith College, Northampton, MA USA; 5 Abbott Nutrition R&D, 3300 Stelzer Road, Columbus, OH USA; 6 Present address: BIO-CAT, 9117 3 Notch Rd, Troy, VA 22974 USA

**Keywords:** Skeletal muscle, Sarcopenia, Proteomics, Diet, Omega-3, Aging

## Abstract

**Background:**

Dietary fish oil (DFO) has been identified as a micronutrient supplement with the potential to improve musculoskeletal health in old age. Few data are available for effects of DFO on muscle contractility, despite the significant negative impact of muscle weakness on age-related health outcomes. Accordingly, the effects of a DFO intervention on the contractile function and proteomic profile of adult and aged in an animal model of aging were investigated.

**Methods:**

This preliminary study evaluated 14 adult (8 months) and 12 aged (22 months) male, Sprague-Dawley rats consuming a DFO-supplemented diet or a control diet for 8 weeks (7 adult and 6 aged/dietary group). Animal weight, food intake and grip strength were assessed at the start and end of the FO intervention. In situ force and contractile properties were measured in the medial gastrocnemius muscle following the intervention and muscles were processed for 2-D gel electrophoresis and proteomic analysis via liquid chromatography with tandem mass spectrometry, confirmed by immunoblotting. Effects of age, diet and age x diet interaction were evaluated by 2-way ANOVA.

**Results:**

A significant (*P* = 0.022) main effect for DFO to increase (~ 15%) muscle contractile force was observed, without changes in muscle mass. Proteomic analysis revealed a small number of proteins that differed across age and dietary groups at least 2-fold, most of which related to metabolism and oxidative stress. In seven of these proteins (creatine kinase, triosephosphate isomerase, pyruvate kinase, parvalbumin, beta-enolase, NADH dehydrogenase and Parkin7/DJ1), immunoblotting corroborated these findings. Parvalbumin showed only an effect of diet (increased with DFO) (*P* = 0.003). Significant age x diet interactions were observed in the other proteins, generally demonstrating increased expression in adult and decreased expression aged rats consuming DFO (all *P* > 0.011). However, correlational analyses revealed no significant associations between contractile parameters and protein abundances.

**Conclusions:**

Results of this preliminary study support the hypothesis that DFO can enhance musculoskeletal health in adult and aged muscles, given the observed improvement in contractile function. The fish oil supplement also alters protein expression in an age-specific manner, but the relationship between proteomic and contractile responses remains unclear. Further investigation to better understand the magnitude and mechanisms muscular effects of DFO in aged populations is warranted.

## Introduction

Impairment of muscular function has significant negative consequences for older adults, as age-associated muscle weakness contributes to multiple chronic medical conditions [1], mortality [2], loss of independent function [3], frailty and risk of falls [4, 5]. In 2004, it was estimated that age-related loss of muscle function accounted for 1.5% of total U.S. annual healthcare expenditures (~$54 billion/yr in 2019 dollars) [6]. A more recent estimate indicated that muscle weakness in older adults in the UK accounted for an additional £2.5 billion in annual costs above older adults without weakness [7]. As the geriatric populations of many western countries are increasing, strategies for maintaining muscle function in old age have become a health-care priority.

In addition to exercise- and pharmacologically-based interventions for improving aged muscle function, dietary supplements may hold some promise as alternative or adjunctive strategies. One such dietary intervention that has received substantial attention in recent years is dietary fish oil (DFO). Multiple benefits, many linked to anti-oxidant and anti-inflammatory action, have been ascribed to DFO in older populations [8, 9]. Of note, though increased longevity has not been linked to DFO, a recent scoping review identified DFO as one of only 16 micronutrient supplements with the potential to improve musculoskeletal health in old age [10]. Indeed, a large retrospective cohort study found that fatty fish consumption was an independent predictor of grip strength in men and women [11]. In addition DFO does not appear to interfere with the benefits of exercise to enhance muscle strength, in contrast to some supplements which may blunt various exercise benefits [12–15]. A number of anti-inflammatory effects known to reduce oxidative injury have been ascribed to DFO and it has therefore been suggested that it reduces the age-associated accumulation of markers of inflammation and oxidative injury in skeletal muscle, where they may contribute to loss of mass and force production [16–18].

Because aging is multifactorial, investigators have turned to genomic, metabolomic and proteomic analyses as a means for obtaining a broad overview of age-related changes in muscle [19–22]. A number of these studies have suggested that dysregulation of oxidative metabolism may contribute to age-related muscle impairments [22–24]. At least one group has used this methodology to discriminate the proteomic profile of aging from that more specifically associated with sarcopenia (the age-related decline in muscle mass) [20]. However, it has become increasingly clear that loss of muscle mass alone does not account for the muscle weakness that accompanies increasing age [25]. Further study is needed to better characterize these mass-independent, molecular mechanisms of weakness, so that they can be targeted for potential interventions. To our knowledge, no detailed proteomic study of the interactive effects of aging and DFO has been reported. Accordingly, the purpose of the present study was to compare the effects of an 8-week DFO intervention on the muscle function and proteomic profile of adult and aged rats, and evaluate the hypothesis that DFO could improve muscle contractile function and that the enhancements would be associated with proteomic changes. Given the significant impact muscle weakness and injury have on a myriad of age-related health outcomes, such findings would have important long-term implications for concepts, treatments, and preventative interventions in several fields, including: geriatric medicine, physical medicine and rehabilitation and nutrition.

## Methods

### Ethical approval

Animal use and all procedures were approved by the Ohio University Institutional Animal Care and Use Committee, and the “Principles of laboratory animal care” (NIH publication No. 86–23, revised 1985) were followed throughout the study. These guidelines are consistent with the journal guidelines and comply with its animal ethics checklist.

### Experimental animals

Adult (Ad; 6 months at receipt; *n* = 14) and aged (Ag; 20 months at receipt, *n* = 12), male Sprague Dawley rats were purchased from Harlan (now ENVIGO, Indianapolis, IN). This strain has been found to exhibit a pattern of sarcopenia similar to that observed in aging humans [20]. All rats were housed individually in an environmentally controlled facility (12–12 h light–dark cycle, 22 °C) at Ohio University (Athens, OH) and allowed to acclimate to the animal facility for 2 weeks, with ad libitum access to purified diet (American Institute of Nutrition rodent diet AIN-93 M; 12.4% protein; 68.4% carbohydrate; 4.1% fat by weight) and water. These animals were part of a study that also involved injuring one of the hindlimbs. Data regarding the responses of the injured muscles have been reported elsewhere [26]. In the present paper, only data from the uninjured limb are presented. The proteomic and specific contractile analyses here have not been published previously.

### Dietary intervention

Following the two-week acclimation to purified diet, half the rats (7 Ad and 6 Ag) were assigned to an 8-week control (Ctl) diet (i.e., continued AIN-93 M). The remaining animals were placed on an 8-week experimental diet AIN-93 M diet formulated with fish oil (FO) at a concentration of 33.65 g per 1 kg AIN-93 M diet (soybean oil comprised the remaining amount of fat for total 40 g fat per 1 kg diet, or 4% of total weight). The FO contained 28.4% eicosapentaenoic acid (EPA) and 12.7% docosahexaenoic acid (DHA). This provided an estimated FO dose of ~ 1.22 g/kg bw/day or EPA dose of 0.35 g/kg bw/day, based on previous studies of aged S-D rats with average body mass of 550 g and food intake ~ 20 g/day [27, 28]. This high dose of FO was chosen for two reasons. It is expected to deliver a metabolically-corrected dose of EPA that is effective at mitigating muscle loss in human cancer cachexia (3 g EPA per day [29]). Second, a previous study of rats found dose was well tolerated and enriched the skeletal muscle long chain polyunsaturated fatty acids (PUFAs), compared to rats that were fed control diet without FO [30]. Diets were color-coded to facilitate blinding of dietary assignment, and were prepared by Research Diets Inc. (New Brunswick, N.J., USA). The FO (LOT#141107D1) was manufactured by NISSUI (Tokyo, Japan) and provided by Abbott Nutrition (Columbus, Ohio, USA). The Ctl diet contained a percentage of fat (soybean oil) equal to that of the DFO diet [26]. Both groups of animals were allowed ad libitum access to food and water.

### Muscle function

Volitional muscle function was assessed 24 h prior to the start of the dietary intervention and again 24 h prior to injury. Bilateral forelimb grip strength, conducted as previously described [23], using a Columbus Instruments dual sensor 1027DR grip strength meter (Columbus, OH, USA) with a triangle bar attachment. The peak forces of 5 trials to failure were averaged for our measure of grip strength, and were expressed in absolute terms and relative to body mass.

At the conclusion of the 8-week dietary intervention, contractility of the both medial gastrocnemius (MG) muscles was evaluated in situ (48 h post injury as described previously [26]), with only the data from uninjured limb analyzed here. The MG was studied as it has been confirmed to exhibit sarcopenia in Sprague-Dawley rats [31]. Prior to contractile testing, animals were anesthetized (Ketamine + Xylazine; 40 + 10 mg kg ^− 1^ body mass), then mounted in a rigid frame that securely immobilizes the leg and pelvis with the distal tendon of the MG clamped in series with a force transducer. Contractile function in response to supramaximal electrical stimulation was assessed with a single pulse (twitch) and a 100 *Hz*, 500 ms train (tetanic). In addition to the peak forces generated during stimulation, twitch contractile properties (time-to-peak tension (TPT) and half-relaxation time (1/2RT) and maximum rates of tetanic force development (RFD) and relaxation (RFR) (absolute and normalized to contractile force) in response to tetanic stimulation, and determined muscle cross-sectional area (CSA) to calculate muscle quality, were all determined as previously described [32]. Following contractile testing, muscles were dissected, blotted dry and weighed. While still anesthetized, rats were euthanized by intracardiac administration of anesthetic per approval by the Ohio University Institutional Animal Care and Use Committee. Some of the muscle tissue was used in experiments on which we have reported elsewhere [26]. A portion of the muscle that was snap frozen in liquid nitrogen, was stored at − 80 °C and shipped on dry ice to the Smith College Center for Proteomics (Northampton, MA USA) for proteomic analyses. Personnel conducting these analyses were blinded to group assignment.

### 2D gel electrophoresis

Frozen muscle samples (*n* = 7 for each adult dietary group: and 6 for each aged dietary group) were weighed, minced on cold glass, homogenized in buffer at 4 °C (10 mM sodium phosphate, pH 7.2, 2 mM EDTA, 10 mM NaN_3_, 120 mM NaCl, 2% NP-40, plus protease and phosphatase inhibitors (ThermoFisher Scientific, MA, USA # 78442)), incubated on ice for 1 h, and centrifuged at 14,000 x g for 30 min [23]. Both the supernatant and pellet fractions were saved, and supernatants, representing the sarcoplasmic fraction, were analyzed in the present study. Protein content of the homogenates was estimated using the method of Lowry, modified for detergent compatibility [33].

Protein was twice-precipitated with acetone from the homogenates and 800 μg protein were suspended in urea-CHAPS buffer (8 M urea, 50 mM DTT, 4% CHAPS, 0.2% pH 5–8 carrier ampholytes, 0.0002% Bromophenol Blue) and loaded onto 11-cm, pH 5–8 IPG strips (Bio-Rad Labs) and focused 40,000 V-Hrs [34]. After focusing, immobilized pH gradient (IPG) strips were equilibrated in a denaturing SDS buffer (6 M urea, 2% SDS, 0.05 M Tris/HCl, 20% glycerol) and alkylated, embedded onto 11-cm 10.5–14% Tris-HCl gels (Bio-Rad Labs), and electrophoresed at 120 V [35]. Gels were stained with Coomassie Brilliant Blue R-250 and imaged using QuantityOne software (v 4.6, Bio-Rad Labs; VersaDoc Scanner, Model 4000, Bio-Rad Labs) and cropped to 122.1 mm × 71.3 mm size for uniformity.

### Proteomic analysis

The 2D gel images (*n* = 5 for each group) were loaded into a match set using PDQuest software (v 8.0.1, Build O55, Bio-Rad Labs, CA, USA). These high-resolution gel images provided highly reproducible match sets for the group comparisons. Large protein spots displaying multiple peaks (identified as the same protein by liquid chromatography with tandem mass spectrometry (LC-MS/MS)) were electronically combined into one spot, and streaks and speckles were removed. Gels were normalized using PDQuest’s local regression model to correct for loading variation. Spot matching was performed automatically by PDQuest, with manual adjustments and removal of spots within the dye front and unresolved side columns. Spots that showed a ± ≥2-fold difference to the respective control (i.e., Ad Ctl vs. Ad DFO) based on quantitative 2D-gel comparisons were excised using an ExQuest Spot Cutter (Bio-Rad Labs), digested with trypsin (In-Gel Tryptic Digest Kit, ThermoFisher Scientific, MA, USA) and de-salted with C-18 columns (Pierce). Proteins from the spots were identified by nanoLC mass spectrometry. Samples (5 μL) were loaded onto an Acclaim PepMap 100 C18 column (3 μm particle size, 75 μm dia, 150 mm long, Thermo Scientific), eluted at 300 nL min^− 1^ over a 40-min 2–50% acetonitrile gradient with 0.1% formic acid using an EASY nLC-1000 HPLC (ThermoFisher Scientific, MA, USA) coupled online to a LCQ Deca XP Max ion trap mass spectrometer (Thermo Electron Corporation, FL, USA), equipped with a Nanospray I source. MS1 scans were recorded between 400 and 1400 m/z, with the 3 most intense ion peaks in each MS1 scan (30.0 s dynamic exclusion enabled) isolated for MS2 fragmentation by collision-induced dissociation (collision energy set to 29). The resultant proteins (Proteome Discoverer 1.4, ThermoFisher Scientific, MA, USA) were grouped by general function into KOG groups (EuKaryotic Orthologous Groups, https://www.ncbi.nlm.nih.gov/COG/).

### Immunoblotting

Proteins representative of various KOG groups that were identified by 2D gel analysis (above) as showing greater than 2-fold differences between groups were selected for quantitative immunoblot validation. Immunoblotting, including stripping and reprobing, was performed as reported previously [33] using validated commercial antibodies (Table 1). Integrated pixel intensities of bands were normalized to the Glyceraldehyde 3-phosphate dehydrogenase (GAPDH) loading control, which did not change with age and diet per proteomic analysis. Values (including GAPDH) were further adjusted for intensities of calibrated protein standards loaded on each gel to account for blot-to-blot variation in transfer efficiency.

**Table 1:** Antibodies used in immunoblots

Protein	Supplier and Catalog Number	Primary Dilution	Secondary Dilution	Uniprot Accession Number
Creatine Kinase - M	PT^1^ 15,891–1-AP	1:10000	1:10000^4^	P00563
Triosephosphate Isomerase - 1	PT^1^ 10,713–1-AP	1:4000	1:5000^4^	P00939
Beta-Enolase	AB^2^ ab96334	1:2500	1:5000^4^	P13929
Pyruvate Kinase – M2	PT^1^ 60,268–1-Ig	1:10000	1:10000^5^	P52480
NADH Dehydrogenase	PT^1^ 15,301–1-AP	1:2000	1:5000^4^	P19404
PARK7/DJ1	AB^2^ ab18257	1:2000	1:5000^4^	Q99497
Parvalbumin	AB^2^ ab11427	1:2500	1:5000^4^	P02625
GAPDH	MS^3^ MAB374	1:25000	1:25000^5^	P46406

^1^Proteintech

^2^Abcam

^3^Millipore-Sigma

^4^Millipore-Sigma 401,393 [goat anti-rabbit IgG-HRP]

^5^SantaCruz sc-2005 [goat anti-mouse IgG-HRP]

### Statistical analysis

A 2-way (Age X Diet) ANOVA was used to analyze the majority of the data. Grip strength was assessed via a 3-way (Age X Diet X Time) repeated-measures ANOVA, with time as a repeated factor. Normality of data was determined via Shapiro-Wilk tests. If testing indicated a potential violation of normality, a non-parametric test was also performed. As the ANOVA is robust to violations of normality, if the nonparametric confirmed the result of the ANOVA (i.e., *P* < 0.050 or > 0.050), we report the result of the ANOVA. If the non-parametric test gave a different result, we report the non-parametric result. As this was a preliminary, exploratory study, effects and interactions that approached, but did not reach, significance (0.100 > *P* > 0.050) are presented with associated effect sizes (partial η^2^). Where appropriate, post-hoc comparisons were made using Fisher’s LSD test. Further exploratory Spearman correlational analyses were conducted to assess the relationships between select muscle function variables and abundance of proteins as determined by immunoblotting, both for the total sample and separately for the Ad and Ag animals (see below for specifics).

## Results

### Body mass and food intake

Body mass was assessed at the start and the completion of the dietary intervention. Effects of time and age were observed, but no effect or interactions related to diet were found (Table 2). As reported elsewhere [26], the food consumption of DFO groups over the 8 weeks corresponded to a dose ~ 1.15 and 1.00 g DFO/kg body mass/day for the young and old rats, respectively. Old rats were heavier than young rats and both groups gained weight over time. Food consumption showed a significant effect of time and an age X time interaction. Over time, rats consumed less food, and with greater decline the aged animals. When normalized to body mass, food consumption still exhibited a main effect of time and age, but the age X time interaction disappeared.

**Table 2:** Body Mass and Food Consumption

	Adult		Aged	
	Ctl (*n* = 7)	DFO (*n* = 7)	Ctl (*n* = 6)	DFO (*n* = 6)
Week 1 Body mass (g) ^*T, A*^	491.0 ± 18.0	500.8 ± 14.5	544.5 ± 19.2	553.0 ± 23.3
Week 8 Body mass (g)	517.0 ± 19.4	535.0 ± 16.8	567.5 ± 19.0	578.0 ± 19.9
Week 1 Food disappearance (g/week) ^*T, X*^	125.1 ± 6.8	132.3 ± 14.4	136.7 ± 10.7	138.3 ± 6.8
Week 8 Food disappearance (g/week)	116.7 ± 4.1‡	120.4 ± 4.7†	110.8 ± 8.3†	114.3 ± 4.8†
Week 1 Food disappearance/body mass ^*T, A*^	0.264 ± 0.006	0.254 ± 0.006	0.251 ± 0.019	0.251 ± 0.012
Week 8 Food disappearance/body mass	0.227 ± 0.013	0.230 ± 0.010	0.195 ± 0.011	0.199 ± 0.009

Data represent means ± SE

*T* significant effect of time, *A* significant effect of age, *X* significant age X time interaction

† = significantly different from week 1 *P* < 0.050

‡ = significantly different from Week 1, *P* < 0.010

### Muscle function and morphology

**Fig. 1:**
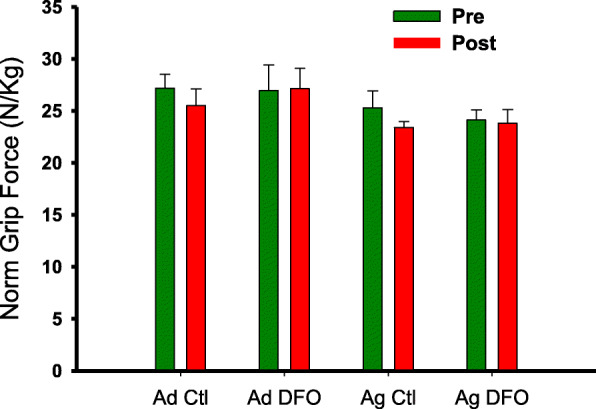
Mean (Ad, *n* = 14 (7 Ctl, 7 FO); Ag, *n* = 12 (6 Ctl, 6 FO), ±SE) bilateral forepaw grip strength normalized to body mass. Pre = prior to initiating dietary intervention; Post = at completion of 8-wk dietary intervention

Effects of age and diet on muscle force, size and contractile properties are summarized in Table 3. Significant effects of age were present for twitch and tetanic force, as well as MG mass and CSA, with reduced values in the aged rats. The peak rate of absolute tetanic force relaxation also exhibited a significant reduction with age. A significant increase in the optimal length for twitch force production (*l*_*opt*_) was also detected. A significant effect of diet was found for tetanic force and muscle quality, with higher values in the FO groups. The main effect for diet on twitch force and muscle quality did not achieve statistical significance (*P* = 0.064; η^2^ = 0.147; *P* = 0.091; η^2^ = 0.119). Significant age x diet interactions were found for 1/2RT and nRFR, both of which showed a general pattern of DFO slowing relaxation in adult animals, but increasing it in older animals. A similar trend for changes in TPT did not achieve statistical significance (*P* = 0.081, η^2^ = 0.160). The significant interaction for *l*_*opt*_ was driven by the observation that all groups had significantly shorter lengths than the aged rats on the Ctl diet. Forearm grip strength exhibited no significant effects or interactions over time. When normalized to body mass, there was a trend for grip strength to decline less in the FO groups over time (Fig. 1), but this effect did not reach significance (Time x Diet interaction, *P* = 0.089, η^2^ = 0.138).

**Table 3:** Muscle Function and Morphology

	Adult		Aged	
	Ctl (*n* = 7)	DFO (*n* = 7)	Ctl (*n* = 6)	DFO (*n* = 6)
Twitch Force (N) ^*A*^	4.36 ± 0.32	5.53 ± 0.38	3.78 ± 0.40	4.04 ± 0.35
Tetanic Force (N) ^*A, D*^	12.76 ± 0.44	14.87 ± 0.88	10.02 ± 0.86	11.65 ± 0.79
Twitch Muscle Quality (N/cm^2^)	5.01 ± 0.26	6.42 ± 0.51	5.35 ± 0.49	5.30 ± 0.40
Tetanic Muscle Quality (N/cm^2^) ^*D*^	14.68 ± 0.47	17.25 ± 1.14	13.82 ± 0.60	15.28 ± 0.76
Muscle Mass (g) ^*A*^	1.33 ± 0.05	1.35 ± 0.05	1.15 ± 0.07	1.19 ± 0.04
Muscle CSA (cm^2^) ^*A*^	0.87 ± 0.03	0.87 ± 0.03	0.71 ± 0.04	0.76 ± 0.03
TPT (ms)	31.8 ± 1.7	35.6 ± 2.5	35.5 ± 2.8	30.8 ± 1.3
1/2 RT (ms) ^*X*^	24.3 ± 2.1^**a**^	30.3 ± 3.5	40.3 ± 9.6	21.0 ± 0.8^**a**^
RFD (mN/ms)	311.7 ± 26.4	340.4 ± 70.9	252.0 ± 27.9	327.8 ± 30.8
RFR (mN/ms) ^*A*^	271.3 ± 19.1	270.2 ± 31.5	149.1 ± 37.2	236.9 ± 9.2
norm RFD (%/ms)	2.5 ± 0.2	2.3 ± 0.2	2.7 ± 0.3	2.9 ± 0.4
norm RFR (%/ms) ^*X*^	2.2 ± 0.2^**a**^	1.8 ± 0.1	1.5 ± 0.2	2.0 ± 0.2
Twitch:Tetanus	0.34 ± 0.01	0.37 ± 0.04	0.39 ± 0.03	0.34 ± 0.02
*l*_opt_ (mm) ^*A, X*^	32.5 ± 0.1^a^	32.8 ± 0.3^**a**^	34.5 ± 0.1	33.0 ± 0.3^**a**^

Data represent means ± SE

^a^significantly different from Aged Ctl

*A* significant effect of age, *D* significant effect of diet, *X* significant interaction

*CSA* cross-sectional area, *TPT* time to peak twitch force; *½ RT* half-relaxation time of twitch force, *RFD* rate of tetanic force development, *RFR* rate of tetanic force relaxation; norm, *RFD* rate of tetanic force development normalized to peak tetanic force, *RFR* rate of tetanic force relaxation normalized to peak tetanic force, *twitch:tetanus* ratio of peak twitch to peak tetanic force, *l*_opt_ optimal length for twitch force production

### Protein markers

#### Proteomic analysis

**Fig. 2:**
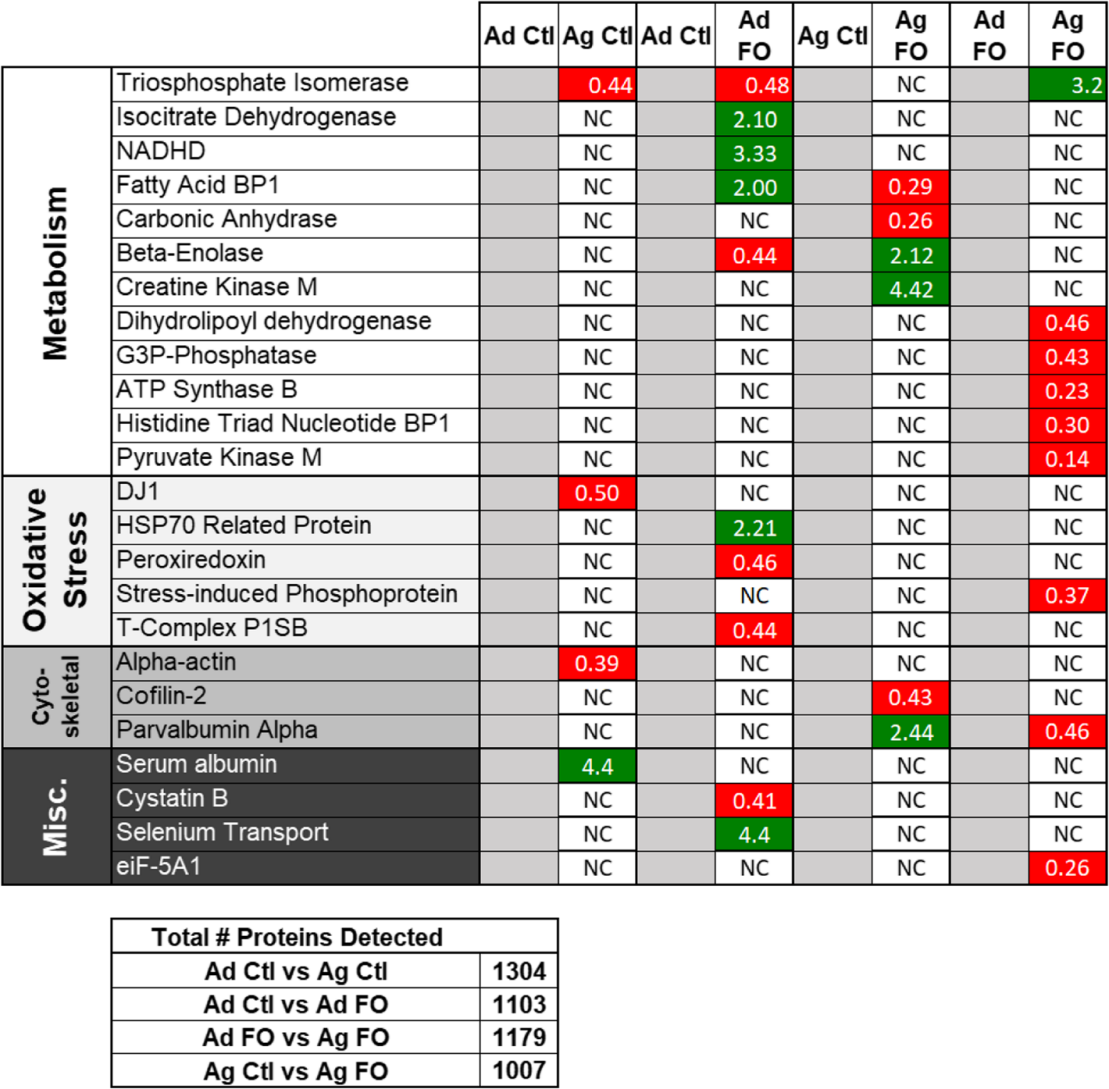
Differential protein expression as a result of aging and DFO. Proteins that differed in expression by greater than or equal to two-fold from the respective control based on quantitative digital 2D gel analyses were identified by nanoLC mass spectrometry and grouped by general function. Red indicates a 2-fold or more reduction, green indicates a 2-fold or more increase. NC = 2-fold change was not observed

**Fig. 3:**
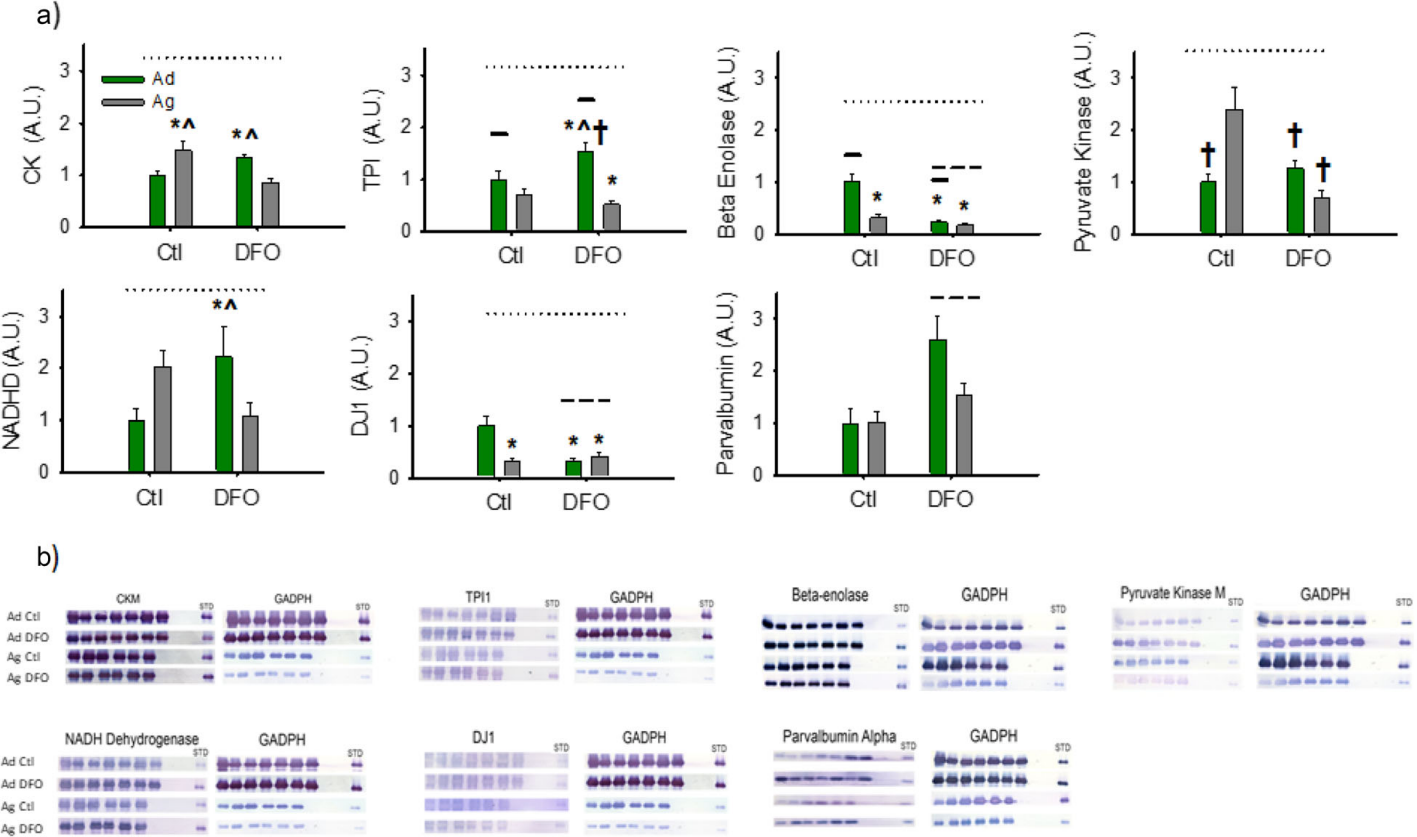
**a**) Mean (Ad, *n* = 14 (7 Ctl, 7 FO); Ag, *n* = 12 (6 Ctl, 6 FO), ± SE) protein abundance (arbitrary units, A.U.), normalized to Adult Ctl diet group, by Western blot for CK, TPI, Beta Enolase, PK, NADHD, DJ1 and Parvalbumin. Solid line = Significant main effect of age; Dashed line = Significant main effect of diet; Dotted Line = Significant age X diet interaction; * = significantly different from Ad Ctl group; ^ = Significantly different from Ag FO group; † = Significantly different from Ag Ctl Group. **b**) Representative immunoblots with the identical standard for corresponding proteins in A along with the corresponding GAPDH loading blots

The total number of proteins detected in the 2-D gels for each group (> 1000 for each) is reported in Fig. 2. Twenty-four proteins exhibited more than 2-fold differences between age or diet groups from the 2D gel image analyses. There were significant differences between all groups, although there were fewer changes between some groups than others. In the comparison between Ad Ctl and Ag Ctl there were three downregulated, and one upregulated, proteins as a function of age. More generally affected by age was the Ad DFO versus Ag DFO group, where eight proteins were downregulated with diet from Ad DFO to Ag DFO. Diet affected young rats significantly as seven proteins were upregulated with diet, and three were downregulated from Ad Ctl to Ad DFO. The changes with diet between Ag Ctl and Ag DFO were more varied, where three proteins were upregulated and three proteins were downregulated. Generally, these differences were confirmed by subsequent immunoblot validation experiments (Fig. 3).

#### Immunoblotting

An age X diet interaction was observed for all of the proteins assessed via immunoblot, except for parvalbumin, which exhibited a main effect of diet only (Fig. 3). As described in Section 2.8, the panels for DJ1 and PK describe the results of non-parametric analysis. The other panels reflect the results of the 2-way ANOVA. The general trend was for DFO to increase protein abundance in adult animals, while it either reduced abundance or induced minimal changes in aged animals. Exceptions were DJ1, where the pattern was reversed, parvalbumin, which increased with DFO in both adult and aged animals, and beta enolase where a much greater decrease was observed in adults.

### Correlational analyses

**Fig. 4:**
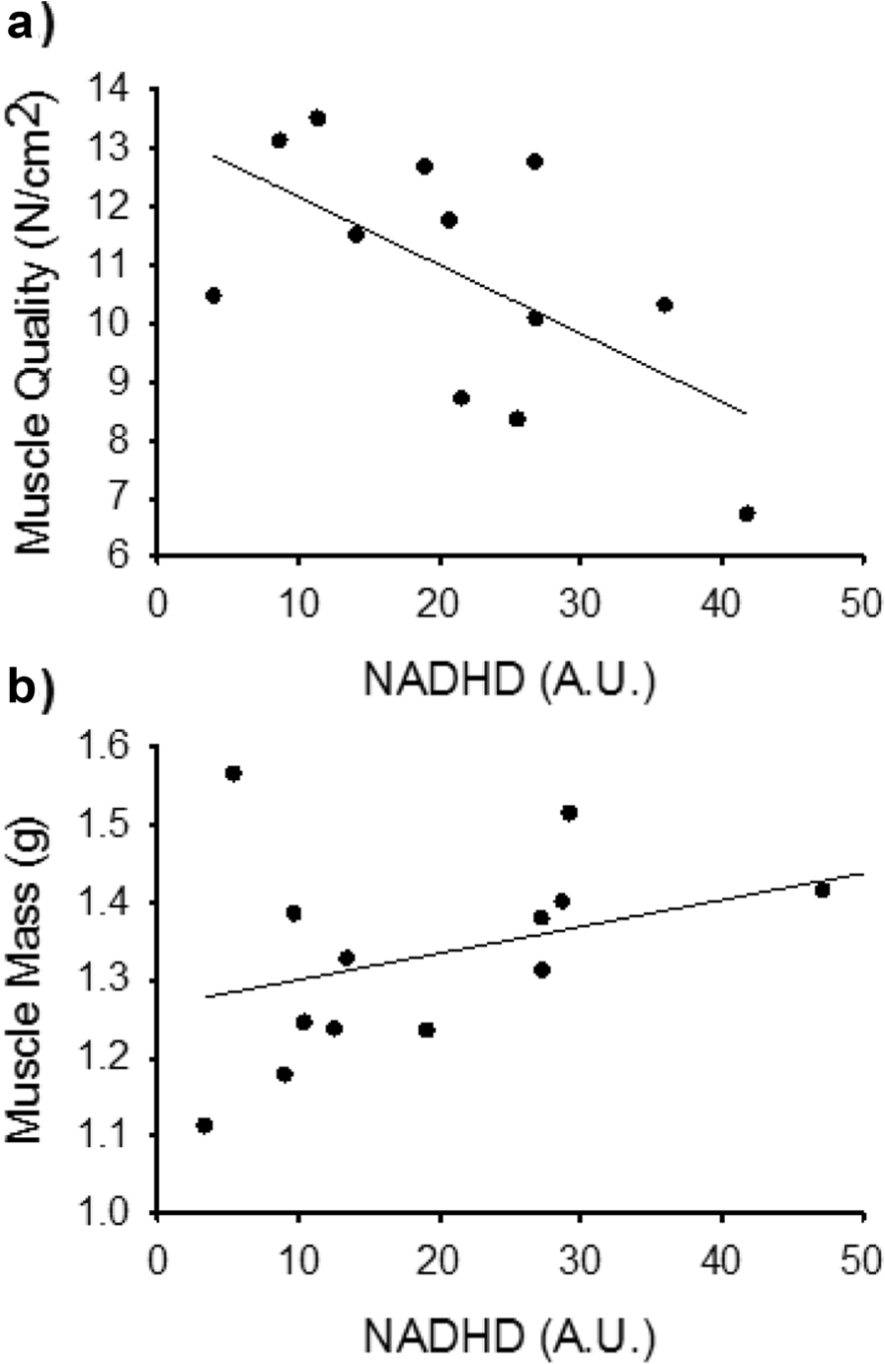
Scatterplots showing relationships between NADHD abundance (arbitrary units, A.U.) and muscle functional and morphological parameters, with lines of best fit. **a**) Ag group NADHD vs. Tetanic Force (r_s_ = − 0.648, *P* = 0.043). **b**) Ad group, NADHD vs. Muscle Mass (r_s_ = 0.503, *P* = 0.067)

Spearman correlations were conducted to explore associations between the muscle function and morphology variables exhibiting effects of DFO and/or age (Table 2) and protein abundances determined by immunoblot. When analyzing the entire sample, no significant associations were observed. When Ad and Ag were analyzed separately, some interesting qualitative differences emerged (Fig. 4). For example, in the Ag group, the muscle force and size-related parameters tended to exhibit negative associations with the markers of energy metabolism, reaching significance for NADHD and tetanic force (r_s_ = − 0.648, *P* = 0.043). In contrast these associations tended to be positive for the Ad animals, with the correlation between NADHD and muscle mass nearing significance in this group (r_s_ = 0.503, *P* = 0.067). The Ad group also exhibited a significant association between parvalbumin and tetanic muscle quality (r_s_ = 0.587, *P* = 0.027). For the contractile properties, both Ad and Ag rats manifested an association between beta-enolase and a measure of slowed force relaxation. For the Ad, beta-enolase was positively associated with twitch 1/2RT, (r_s_ = 0.665, *P* = 0.009), while the Ag group exhibited a negative association between the protein abundance and the absolute tetanic RFR (r_s_ = − 0.690, *P* = 0.058), though only the Ad group effect was significant. Thus increased beta-enolase was associated with an index of slower force relaxation in both Ad and Ag. Absolute RFR was also positively associated with parvalbumin abundance in the Ag, but not the Ad, group.

## Discussion

This study of DFO in adult and aged rats presents new contractile and proteomic data from an earlier study evaluating the effects of DFO on muscle injury. Consistent with a recent scoping review identifying DFO as a micronutrient with potential to improve musculoskeletal health in old age [10], DFO enhanced several aspects of muscle contractility, though the effect was more robust in adult than in aged rats (Table 3). From a proteomic standpoint, dietary DFO had clear effects on sarcoplasmic protein expression, including an interaction with aging whereby DFO supplementation increased abundance in adult and decreased it in aged rats, at least for the bulk of the proteins examined. However, no obvious links between proteomic and contractile changes due to aging or DFO were uncovered.

### Contractile responses

The principal effect of dietary DFO on muscle function was increased tetanic force production without an effect on muscle mass. As both human and animal models indicate that age-related muscle weakness typically cannot be explained simply by loss of mass [25, 32] and the hypertrophic response of aged muscle is often blunted [36, 37], there is a need for interventions that target aged muscle quality (force/unit muscle tissue). The present results suggest that DFO might be effective in this area, though further work is needed to confirm these preliminary findings. Thus, an enhancement of aged muscle contractile function without an increase in mass was not unexpected. However, it was somewhat surprising that the contractile benefits of DFO were greater in adult animals without a gain in muscle mass. One might expect that DFO would be less effective in younger animals that exhibit no real deficits that could account for impaired muscle quality (e.g., excitation contraction coupling, neuromuscular transmission [25]). The reduced contractile enhancement in aged rats may indicate that the effects of DFO on contractility require an additional stimulus (e.g., exercise) to manifest optimally in aged muscles [38]. As volitional physical activity is known to decline in aging rats [23, 39], an age-related reduction in cage activity could have lessened the effects of DFO. This hypothesis is consistent with a recent clinical trial of DFO and exercise in older women [14] and a comparative effectiveness study of resistance exercise and DFO [40]. In contrast, an increase in the resting metabolic rate (RMR) of sedentary older women has been reported [41], while a more recent study from the same group found no change in RMR in healthy, active older adults [42].

In addition, the in situ stimulation protocol, though it bypasses the central nervous system, is still dependent on neuromuscular junction and motor neuron functionality. An enhancement in contractile sensitivity of rodent smooth muscle (i.e., ileum) to acetylcholine has been reported [43], and such a mechanism could be at work here. Impairments of the neuromuscular junction (NMJ), independent of muscle mass, have been reported in aged rat muscle [44, 45], and if DFO enhances sensitivity to acetylcholine in skeletal muscle as it does in smooth muscle, it might partially correct this deficit. It seems less likely that such a mechanism could account for the effect of DFO on the adult rats, given that the safety factor for NMJ transmitter release is believed to be quite high in a young, healthy system. It could be that DFO enhances sarcoplasmic reticulum Ca^2+^ release, which has been shown to be impaired in aged rats [32, 39] and could also enhance muscle quality. However, earlier work indicates that increases in Ca^2+^ release that occur with DFO do not reach statistical significance, though the study may have been underpowered to detect such an effect [26]. Differences in specific tension associated with isoforms of myosin heavy chain (MHC) [46] are unlikely to explain the contractile differences, as our previous work found no change in MHC with DFO [26]. An alternative possibility is that DFO increases myosin light chain (MLC) phosphorylation, the primary mechanism for contractile potentiation in skeletal muscle [47]. As smooth muscle contraction is highly dependent on MLC phosphorylation [48], such a mechanism could possibly account for both our results and those described in smooth muscle, though this is highly speculative and has not yet been investigate.

### Proteomics

The proteomic and immunoblot analyses here indicate several differences in the response of adult and aged muscles to DFO. All of the energy metabolism proteins we evaluated by immunoblotting exhibited an age X diet interaction. Generally, DFO increases the abundance of proteins for substrate-level phosphorylation (CK), glycolysis (PK, TPI) and mitochondrial Complex I electron transport (NADHD) in Ad rats, while it decreases these proteins in the Ag rats (Figs. 3 and 4). Increased energy metabolism is often reported with interventions that improve muscle function in aged and adult subjects [23, 49–51]. In addition, accumulation of glycolytic intermediates (e.g., pyruvate, glycerol 3-phosphate) has been reported in aged vs. adult muscles, suggesting impairments in at least some aspect of glycolytic activity [19, 22], though such findings may not translate to humans [52]. The improvement in contractile function in the Ag muscles, though less than that seen in the Ad muscles, would seem at odds with the reduced abundance of the metabolic proteins.

Protein abundance may not indicate function, however. The finding of increased CK abundance in Ag Ctl muscles is consistent with other data [53], though CK activity typically exhibits a decline or no change in aged humans [54], suggesting a possible increase in overall CK to compensate for impaired functionality. Similarly, impaired glycolytic function is often reported with aging [51, 54], though declines in enzyme abundance are not necessarily observed, and anerobic ATP provision during contraction is maintained in aged rats and humans [55, 56]. It is possible that, in contrast to the Ad group, post-translational modifications in the Ag muscles [17, 57, 58] might impair function of CK and glycolytic enzymes. This functional loss could lead an accumulation of the proteins, both as a means of attempting to maintain function and because the post-translational modifications could reduce the efficiency of breaking down the proteins. This diminished protein turnover could reduce the specific activity of affected enzymes. Thus, dietary DFO might stimulate protein turnover and/or restore normal levels of post-translational modification and improve protein function without necessarily increasing abundance. A number of studies suggest that DFO can stimulate human muscle protein synthesis [38, 59, 60]. While increased turnover would involve increases in both muscle protein synthesis (MPS) and breakdown (MPB) [61], data on MPB in vivo are much less prevalent than those for MPS. Some studies indicate that DFO inhibits overall MPB, but these mostly involve cultured cells and young animals [62–64]. In contrast, proteolysis of specific proteins has been reported for EPA and DHA, often in pathological states (e.g., cancer) [65–67]. In addition, DFO has been found to blunt glycation and other post-translational modifications in diabetic rats [68, 69], supporting speculation that DFO might alter muscle proteins post-translationally.

Interestingly, beta enolase, also a glycolytic protein, exhibited a greater decrease in adult vs. aged muscles with DFO. However, non-glycolytic functions of enolase related to repression of transcription have recently been identified [70]. Since enolase is not believed to play a major regulatory role (i.e., a rate-limiting step) in glycolysis [71], the changes observed here may be more related to these non-glycolytic functions. Future work could examine changes in beta enolase localization, which has been linked to glycolytic vs. non-glycolytic function [70].

Parvalbumin was positively associated with tetanic muscle quality in the Ad group (which showed the greatest increase in tetanic force). This soluble calcium-binding protein is preferentially expressed in fast-twitch fibers. DFO increased parvalbumin abundance in both aged and adult animals, though only the aged muscle exhibited the expected reduction in twitch 1/2RT. In contrast, adult transgenic mice overexpressing parvalbumin have exhibited no change in contractility [72, 73]. Parvalbumin gene transfer via electroporation does not affect contractility in adult mice but impairs it in aged mice [72]. Thus, the role of parvalbumin in our observation of improved contractility is unexpected and requires further study. It may be that the increased parvalbumin partially blunts the contractile benefits of DFO (via an as yet undetermined mechanism), accounting for the relatively smaller response of aged vs. adult rats.

Perhaps expecting a common mechanism related to changes in old and adult muscles is unjustified. Given the effect of aging across physiological systems, it is possible that DFO influences very different mechanisms that enhance contractility. For example, as noted above, enhancements in glycolytic metabolism in adult animals could potentially enhance contractility. In aged animals, a different mechanism could be at work. Here, DFO significantly changed the optimal length for force development in the aged rats (Table 3). Though highly speculative, this might suggest an improvement in myofilament Ca^2+^ sensitivity. As reduced SR Ca^2+^ release has been reported in aged rats, [26, 32, 39], an increase in Ca^2+^ sensitivity could potentially improve force production in older, but not younger muscles. However, such changes would likely be found in the myofibrillar protein fraction, which we were unable to analyze in the present study due to the exclusion of this fraction by pore size of the isoelectric focusing gel.

### Caveats and considerations

This study examined the uninjured muscles of animals that received a contusion injury to one limb. Use of the contralateral limb as the control in injury studies is common practice, and since all animals were injured, this factor did not differ across groups. However, it does raise the possibility that the results observed here reflect an interaction among age, diet and injury and might have been different in animals that were never injured.

Data suggest that this is unlikely with regard to the contractile parameters evaluated. Although stereotyped experimental injuries can induce divergent locomotor responses [74], the acute nature of the injury (contractile testing and tissue harvest occurred 48 h post-contusion) makes it unlikely that sufficient disuse occurred to affect the contractility of the muscle. Indeed, force production in the uninjured muscles of rats following a single-event mechanical injury to the other limb has been shown to be comparable to control [75], and a recent study of contusion injury [76] reported no changes in organ weight or organ damage markers in animals following injury.

From the standpoint of a systemic effect of injury on the proteomics data, a potential effect seems more likely. While uninjured muscles exhibited no increases in angiogenic and growth factors that were elevated in mice with a blunt muscle injury to the contralateral muscle [77], muscle-specific serum markers of muscle injury have been found to increase following single muscle injury [76]. Thus, the potential for elevated systemic factors (e.g. cortisol, IL-6) to influence muscle protein responses cannot be excluded. Although the injury condition was present for all animals, it is possible that it may have interacted differently across age or dietary groups. Similarly, because the muscles were dissected following the contractile testing, we cannot rule out a potential effect of muscle contraction on the findings. This seems unlikely however, given the brief contractility protocol and rapid freezing that followed.

As noted, the proteomic and immunoblot analyses involved only the soluble sarcoplasmic fraction, and more direct links between proteomic changes and functional muscle enhancement with DFO may be established from the myofibrillar protein fraction. The laboratory is currently pursuing this task.

Although GAPDH is commonly used to normalize immunoblot data, including studies of aged muscle, some concerns regarding its use in aging studies have been raised [78]. However, the proteomic analyses (Fig. 2) were not based on GAPDH normalization and they largely agreed with the immunoblot results (section 2.7). In addition, analyses of the immunoblot data never revealed a main effect of age *without* an accompanying age X diet interaction. Thus, it is unlikely that an effect of age on GAPDH expression could account for the present results.

Dosage of DFO was based on food disappearance, which might overestimate actual consumption, and thus DFO dosage. In addition, we cannot rule out acute metabolic effects of food intake (i.e., post-prandial responses), as we did not block access to food prior to contractile testing and muscle harvest.

Finally, the study involved only male rats. Given the numerous physiological differences between males and females that have been identified, these findings should not be extrapolated to females. Indeed, a recent clinical trial reports enhanced muscle torque in older women, but not men, on a DFO-supplemented vs. a control diet [14]. Thus, greater changes might have seen in with DFO in aged female rats.

## Conclusion

Results of this preliminary study support the hypothesis that DFO can enhance musculoskeletal health in adult and aged muscles, given the observed improvement in contractile function, despite no change in muscle mass. Sarcoplasmic protein expression was also altered in an age-specific manner by DFO. However, the relationship between proteomic and contractile responses remains unclear, and was possibly affected by differential interactions with systemic factors from muscle injury. Further investigation to better understand the magnitude and mechanisms underlying the muscular effects of DFO in aged populations is warranted.

## Supplementary information

**Additional file 1.** We have attached a cleaner version of the supplementary file, with landscape orientation.

## Data Availability

The data that support the findings of this study are available from the corresponding author upon reasonable request.

## Abbreviations

1/2RT: Half-relaxation time
Ad: Adult (8 months)
Ag: Aged (22 months)
ANOVA: Analysis of Variance
CHAPS: (3-((3-cholamidopropyl) dimethylammonio)-1-propanesulfonate)
CKM: Creatine kinase, skeletal muscle form
Ctl: Control Diet
CSA: Muscle cross-sectional area
DFO: Diet with added fish oil supplement
DHA: Docosahexaenoic acid
DJ1: Protein deglycase DJ-1 (Parkinson disease protein 7)
DTT: Dithiothreitol
EDTA: Ethylenediaminetetraacetic acid
EPA: Eicosapentaenoic acid
FO: Fish oil
GAPDH: Glyceraldehyde-3-phosphate dehydrogenase
IPG: Immobilized pH gradient
*l*_opt_: Optimal length
MG: Medial gastrocnemius
NaCl: Sodium chloride
NADHD: Reduced Nicotinamide adenine dinucleotide dehydrogenase (ubiquinone)
NaN_3_: Sodium azide
nRFD: Maximum rate of tetanic force development normalized to tetanic force
nRFR: Maximum rate of tetanic force relaxation normalized to tetanic force
PA: Parvalbumin
PKM: Pyruvate Kinase skeletal muscle form
RFD: Maximum rate of tetanic force development
RFR: Maximum rate of tetanic force relaxation
SDS: Sodium dodecyl sulfate
TBS: Tris-buffered saline
TBS-T: Tris-buffered saline plus Tween20
TPI: Triosephosphate Isomerase - 1
TPT: Time-to-peak tension
Tris: Tris (hydroxymethyl)aminomethane
