# Supplementary material for: Dietary fish oil supplement induces age-specific contractile and proteomic responses in muscles of male rats
Source: Lipids Health Dis. 2020 Jul 9;19:165. doi: 10.1186/s12944-020-01333-4 (PMC7350698; doi:10.1186/s12944-020-01333-4)
Supplement: Supplementary file 1 — Additional file 1. We have attached a cleaner version of the supplementary file, with landscape orientation. [file 12944_2020_1333_MOESM1_ESM.pdf]

Protein	Score	Coverage	Description	MW	pI	Accession	KOG Group
78kDa glucose related protein	606.6	55.96	HSP70 chaperone family	72.3	5.16	P06761	O
Actin, alpha muscle	466.4	53.32	Cell motility protein	42	5.39	P68035	Z
NADH dehydrogenase	285.2	43.95	Mitochondrial ETC	27.4	6.68	P19234	C
Peroxiredoxin	482.8	61.11	Eliminates peroxides	21.8	5.59	P35704	O
Fatty acid binding protein	423.1	84.21	Transprt of fatty acid	14.8	6.33	P07483	I
Cystatin-B	76.01	55.1	Thiol proteinase inhibitor	11.2	6.3	P01041	U
Isocitrate dehydrogenase	654.7	39.34	CAC Step 3, NAD+ reducer	39.6	6.92	Q99NA5	C
Serum albumin	2735	73.36	Regulates blood lipid content	68.7	6.48	P02770	I
T-complex protein 1, subunit B	600	60.19	Folds protein, ATP hydrolysis	57.4	6.46	Q5XIM9	O
Selenium-binding protein 1	249.6	56.36	Intra-Golgi protein transport	52.5	6.57	Q8VIF7	P
Beta-enolase	2575	76.5	Glycolysis, muscle development	47	7.44	P15429	G
Triosephosphate isomerase	2510	93.98	Glycolysis, muscle development	26.8	7.24	P48500	G
Parvalbumin alpha	832.7	73.64	Relaxation after contraction	11.9	5.19	P02625	Z
eIF-5A1	100.4	50	Muscle stem cell differentiation	16.8	5.24	Q3T1J1	T
Glycerol-3-phosphate phosphatase	492.1	38.63	Lipid biosynthesis	34.6	5.35	D3ZDK7	I
ATP synthase subunit d	1317	91.93	Mitochondrial ATP synthase	18.8	6.6	P31399	C

Histidine triad nucleotide BP1	205.3	47.62	tRNA metabolism	13.8	6.87	P62959	T
Pyruvate kinase PKM	485	62.15	ATP synthesis	57.8	7.06	P11980	G
Stress-induced-phosphoprotein 1	425.5	55.8	HSP 70/90 oraganizing protein	62.5	6.8	O35814	O
Dihydrolipoyl dehydrogenase	1094	55.6	Mitochondrial lipid metabolism	54	7.87	Q6P6R2	C
DJ1	571	77.78	Deglycase	20	6.3	Q00LX0	V
Creatin Kinase muscle isoform	4697	70.87	Creatine energy metabolism	43	7.06	P00564	C
Carbonic anhydrase 3 muscle isoform	569.4	58.46	Carbon dioxide hydration	29.4	7.37	P14141	R
Cofilin 2	917.9	74.7	Actin filament regulator	18.7	7.88	M0RC65	Z
